# Preparation of microgel co-loaded with nuciferine and epigallocatechin-3-gallate for the regulation of lipid metabolism

**DOI:** 10.3389/fnut.2022.1069797

**Published:** 2022-12-12

**Authors:** Shengnan Zhu, Weijia Xu, Jun Liu, Feng Guan, Aichun Xu, Jin Zhao, Jian Ge

**Affiliations:** College of Life Sciences, China Jiliang University, Hangzhou, Zhejiang, China

**Keywords:** nuciferine, epigallocatechin-3-gallate, microgel, miRNAs, lipid metabolism genes, gut microbiota

## Abstract

This study aims to enhance the stability and bioavailability of nuciferine (NF) and epigallocatechin-3-gallate (EGCG) by loading NF into liposomes and then incorporating the liposomes and EGCG into porous microgels (NFEG-microgel) prepared with chitosan and proanthocyanidin. Analysis of particle size (0.5–3.0 μm), electron microscopy, rheology, stability, and simulated gastrointestinal release confirmed that the prepared microgels had high encapsulation rate and good stability and release characteristics. Intervention experiments were performed by orally administering NFEG-microgel to high-fat diet rats to evaluate its efficacy and regulatory mechanism for blood lipid metabolism. NFEG-microgel intervention significantly reduced the body weight and serum lipid level, and the mechanism was related to the expression regulation of key genes involved in lipid metabolism and miRNAs (miR-126a-5p and miR-30b-5p) in serum extracellular vesicles. In addition, NFEG-microgel improved the diversity of gut microbiota by enriching short-chain fatty acids (SCFA)-producing bacteria and reducing harmful bacteria, suggesting that it can ameliorate lipid metabolism by regulating the intestinal flora community in rats.

## Graphical Abstract

NFEG-microgel was prepared and intervention experiments were performed by orally administering NFEG-microgel to high-fat diet rats to evaluate its efficacy and regulatory mechanism for blood lipid metabolism. The amelioration of lipid metabolism was relative to regulating the expression of key genes which interacted with miR-126a-5p and miR-30b-5p in serum extracellular vesicles and the intestinal flora community in rats. 
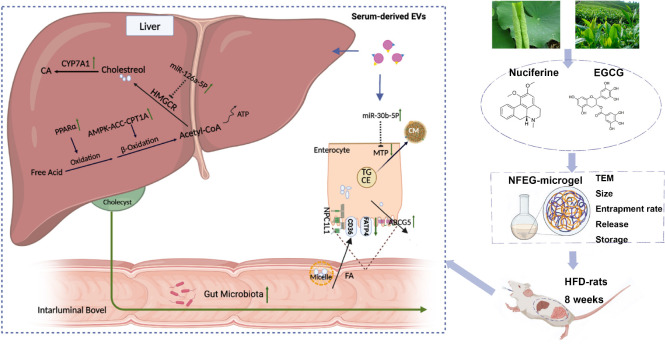

## Introduction

Nuciferine (NF, PubChem CID: 10146) is an aromatic ring-containing alkaloid extracted from *Nelumbo nucifera* leaves and has a wide variety of pharmacological activities, such as lipid lowering (1), anti-inflammatory, antioxidant, anti-liver injury, and anticancer (2). NF also inhibits the accumulation of lipid in 3T3-L1 preadipocytes by regulating the expression of lipid gene (3) and improves the lipid profile in mouse diabetic model by activating peroxisome proliferator-activated receptors-α (PPAR-α)/peroxisome proliferator-activated receptor-γ coactivator-1α (PGC1α) (4). In addition, NF treatment changes the composition of the intestinal microbiota of rats fed with high-fat diet (HFD) (5).

Epigallocatechin-3-gallate (EGCG), the major polyphenolic catechin of green tea, is the most abundant antioxidant catechin accounting for about 50∼80% of total catechins content. Although the biological effects of EGCG have not been completely discovered, it can enhance lipid metabolism, has anti-inflammatory and antioxidant activities, and can reduce tumor incidence. Increasing evidence indicates that EGCG has a wide range of biological activities, including activating AMP-activated protein kinase (AMPK), promoting lipid metabolism, and improving insulin resistance, which has a multipronged preventive and therapeutic effect on non-alcoholic fatty liver disease (NAFLD) (6). In addition, EGCG can improve different types of liver injury (7).

Nuciferine has extremely low water solubility and dispersion, and its oral bioavailability is only 3.9% (8). Its stability, bioavailability, and absorptivity can further decline due to gastrointestinal conditions, which is similar to the trend of the stability and bioavailability of EGCG. In humans, the maximum plasma concentration of EGCG is only 0.15 μM after consuming two cups of green tea (4). The double-layer encapsulation of liposome and microgel is adopted to improve the stability and bioavailability of NF and EGCG. In this process, NF and EGCG are encapsulated simultaneously to play a synergistic role at different points in the metabolic cycle. Microgels (d. 1∼350 μm) are swellable polymer networks with hydrophilic functionalities that allow them to entrap large amounts of water without collapse and are usually prepared by polymer (polysaccharides and/or proteins) cross-linking through ionic, physical, or covalent interactions (9). Microgels have been widely used in the encapsulation of various unstable substances, such as astaxanthin (10) and polyphenols (11), to improve their stability and bioavailability and control their release. *Myrica rubra* leaf proanthocyanidin extract (MLPE) and chitosan are abundant natural materials widely used in the food industry because of their safety and good functional properties. Chitosan coating can increase the stability of liposomes in various biological fluids including simulated gastric fluid (SGF) and simulated small intestinal fluid (SIF), the adhesion of mucosa, and the solubility of drugs (12).

Lipid metabolism is a process of lipid synthesis and degradation in cells, including lipid decomposition, storage for energy and synthesis of structural and functional lipids. Its main function is to transport lipids to the peripheral tissues for use or to transport lipids back to the liver for recycling and removal. And their disorders will lead to the occurrence of many diseases that seriously endangers people’s life and health. According to the functional division, lipid metabolism is roughly composed of three parts: exogenous lipid absorption, endogenous lipid synthesis, and adverse cholesterol transport (13). Lipid metabolism disorders lead to the increase of total cholesterol (TC), triglyceride (TG), and low-density lipoprotein cholesterol (LDL-C), and the decrease of high-density lipoprotein cholesterol (HDL-C) which can induce a variety of diseases such as non-alcoholic fatty liver disease and varying evolutions. The liver is a vital organ of the regulation of lipid steady state. Excessive accumulate of lipid in the liver and overloaded liver cause subsequent series of inflammation, insulin resistance, oxidative stress, mitochondrial dysfunction, and intestinal flora disturbance, in the occurrence and development of NAFLD, resulting in multiple attacks on the liver (14). And the published literatures show that changes in the expression or activity of miR-34a, miR-33, miR-122, and miR-21 are the key mechanisms responsible for the development of NAFLD and its progression toward a serious status (15). Animal studies elucidated that the intestinal microbiota, which is consistently rich in proteobacteria, may have a causal role in NAFLD (16).

In our study, NFEG-microgel was prepared, characterized, and used to prevent and inhibit dyslipidemia or NAFLD. Its regulating mechanism for lipid metabolism disorder was also investigated and clarified.

## Materials and methods

### Materials and instruments

Nuciferine was bought from MedChemExpress Co., Ltd. (Shanghai, China, purity > 98%). Standard EGCG was obtained from Beijing Solarbio Technology Co., Ltd. (Beijing, China, purity > 98%). Chitosan of medium molecular weight was purchased from Sigma–Aldrich (St. Louis, MO, USA, purity > 75%). Lipo3000 transfection reagent was acquired from Thermo Fisher Co., Ltd. TRIzol^®^ Plus RNA Purification Kit and Transcriptor First Strand cDNA Synthesis Kit were bought from Thermo Fisher (Waltham, MA, USA). Dual-luciferase reporter assay system was purchased from Promega Co., Ltd. (Madison, WI, USA).

The following instruments were used in this study: Zetasizer Nano ZS90 (Malvern Instruments Co., Ltd., Malvern, UK), MIKRO 22R centrifuge (Kirchlengern, NRW, Germany), OLYMPUS BX60 fluorescent microscope camera (OLYMPUS Corporation, Tokyo, Japan), transmission electron microscope (TEM) (Hitachi, Tokyo, Japan), and CFX384 multiplex real-time fluorescence quantitative PCR instrument (Bio-Rad, Hercules, CA, USA).

### Manufacturing of nuciferine-loaded liposomes

Nuciferine-loaded liposomes (NF-liposomes) were manufactured by solvent evaporation and ultrasonic homogenization following a previous method (17) with some modifications. In brief, 1 g of soybean phospholipids (Shanghai Taiwei Pharmaceutical Co., Ltd., Shanghai, China) and 0.2 g of cholesterol (Shanghai Titan Chemical Co., Ltd., Shanghai, China) were dissolved in trichloromethane and stirred with magnetic force. After complete distribution, 0.2 g of NF dissolved in methanol was added and the mixture was stirred evenly. The organic solvent was evaporated by rotation (30 rpm) at 30°C to form a light-yellow film, which was then dispersed with 0.05 M phosphate buffered saline (PBS). Ultrasonic crushing was performed in an ice bath for 6 min to obtain a translucent liposome solution. Finally, particle size, zeta potential, and polydispersity index (PDI) were analyzed by Zetasizer Nano ZS90. Encapsulation rate was determined by high-performance liquid chromatography (HPLC).

### Preparation of chitosan-procyanidin microgels loaded with nuciferine and epigallocatechin-3-gallate

Chitosan–procyanidin microgels were prepared following the methods of Zhang et al. (18) and Zou et al. (19) with some modifications. Chitosan was dissolved in 1% acetic acid solution to a concentration of 6 mg/ml. The liposome solution loaded with NF was mixed with chitosan solution, and magnetic stirring was performed for 1 h. MLPE with purity of 70% and average polymerization degree of 6.7 was extracted in our lab. The two concentrations of MLPE (2 and 6 mg/ml) were added to the above solution (EGCG was dissolved in advance so that the final concentration was 2 mg/ml), and magnetic stirring was performed for at least 3 h to obtain NF-EGCG double-encapsulated microgel (NFEG-microgel). After overnight refrigeration, the encapsulation rate and stability of the two concentrations of MLPE microgels were compared. The results showed that the encapsulation rate was high and stable upon the addition of 2 mg/ml MLPE in the microgel. Thus, subsequent microgels were prepared with this concentration.

### Characterization

#### Encapsulation efficiency

After the preparation of microgels, encapsulation efficiency was determined by ultrafiltration centrifugation. In brief, 0.5 ml of NFEG-microgel solution was centrifuged in an ultrafiltration centrifuge tube at 4,000 g for 30 min. The liquid supernatant of the outer tube, which contained unencapsulated NF and EGCG, was obtained. The content of NF and EGCG was determined by HPLC, and the encapsulation efficiency of NFEG-microgel was calculated according to the following formula:


$$\mathrm{Encapsulation}\,\mathrm{efficiency}=\frac{W_{1}-W_{2}}{W_{1}}\times 100\%,$$

where W1 is the total NF or EGCG weight of NFEG-microgel, and W2 is the weight of NF or EGCG in the supernatant.

#### Morphological observation

The sample was first dispersed and dropped onto a carbon coppered grid, and the morphological characteristics of the microgel prepared with chitosan and MLPE were photographed by TEM.

#### Average particle size, polydispersity index, and zeta potential

Particle size was measured by LS13 320 Laser particle size analyzer (Beckman coulter, Brea, CA, USA), and zeta potential, and PDI were analyzed by Zetasizer Nano ZS90 after the sample was dispersed.

#### Rheological property analysis

The rheological properties of samples were determined using Discovery Hybrid Rheometer (TA Instruments, Wilmington, DE, USA) (20). The measured shear rate (τ) increased from 0.01 to 500 s^–1^ and was used to evaluate the viscosity and shear stress of the microgel.

#### Simulated gastrointestinal digestion of NFEG–microgel *in vitro*

In brief, 5 ml of microgel was first digested in 5 ml of SGF with pH of 2.0 for 2 h, followed by the addition of 10 ml of SIF and incubation for 6 h. The pH was adjusted to 7.0 with 0.1 M NaHCO_3_ solution following a previous method with some modifications (11). Incubation was carried out in a shaker under 120 rpm/min and 37°C. The samples were extracted at 0.5, 1, 2, 4, 6, and 8 h, and the same volume of release media was added. SGF composed of 0.9 g of NaCl and 0.14 g of pepsin (1:3,000, Beijing Solarbio Technology Co., Ltd., Beijing, China) was dissolved in 100 ml of 0.1 mol/L HCl aqueous solution at a final pH of 2.0. The simulated SIF was composed of 0.225 g of trypsin (1:250, Hefei Bomei Biotechnology Co., Ltd., Hefei, China) and 1.125 g of pig bile salt (China National Institute for Food and Drug Control, Beijing, China).

#### Storage stability

Nuciferine solution dissolved in 0.5% sodium carboxymethyl cellulose, EGCG solution, and NFEG-microgel were stored at 4°C for 12 weeks at their corresponding concentrations. Their storage stability was evaluated by their apparent appearance and encapsulation rate (21).

### Animal sample collection and processing

Thirty healthy male Wistar rats (180 ± 20 g) with special pathogen free were purchased from SLAC Laboratory Animal Co., Ltd. (Shanghai, China) with animal license number SCXK (Shanghai, China) 2017-0005. The rats were raised in standard conditions (temperature 25 ± 1°C, relative humidity 40–60%, and 12 h light/dark cycles) and had free access to food and water. Their dietary consumption was recoded daily. All animal procedures were permitted by the Laboratory Animal Ethics Committee (2022-005) of China Jiliang University (Hangzhou, China). After a week of acclimatization, the rats were randomly divided into the following six groups with five rats each: blank control (NC), HFD, NF-loaded microgel treatment with high-fat diet (HFD + NF), EGCG-loaded microgel treatment with HFD (HFD + EGCG), NF and EGCG co-loaded microgel treatment with HFD (HFD + NFEG), and empty microgel treatment with HFD (HFD + MG). The NC group received commercial normal diet, and the remaining groups received HFD purchased from Fanbo Animal Feed Biotechnology Co., Ltd. (Shanghai, China) and consisted of 10% lard, 10% yolk powder, 6% casein, 3% maltose, 2% cholesterol, 1.2% premix, and 0.1% cholate. The rats in HFD + NF group intragastrically received NF at 20 mg/kg/day, those in HFD + EGCG group intragastrically received EGCG at 20 mg/kg/day (22), and those in HFD + NFEG group intragastrically received the same NF (20 mg/kg/day) and EGCG (20 mg/kg/day) for 8 weeks (23), The volume of the treatments was 10 ml/kg of rat body weight. The rats in NC and HFD + MG groups intragastrically received the same volume of water and empty microgel, respectively, for 8 weeks. Food intake was recorded daily, and body weight was monitored weekly. Blood samples were collected from the tail every 2 weeks and then centrifugated at 1,500 × g for 15 min at 4°C to separate the serum. The biochemical indexes of the serum were detected using the corresponding kits. Triglyceride (TG), total cholesterol (TC), high-density lipoprotein (HDL-C), and low-density lipoprotein (LDL-C) kits were purchased from Thermo Fisher Co., Ltd. (Waltham, MA, USA). At the end of the experiment, the rats were sacrificed. Blood, liver, small intestine, and cecum contents were obtained and stored at –80°C. The liver was sectioned, preserved in formalin fixative, embedded in paraffin, sectioned at 4 μm, and stained with hematoxylin and eosin (H&E).

### Real-time quantitative PCR analysis

Total RNA of liver and small intestine tissues was extracted using TRIzol^®^ Plus RNA Purification Kit following the manufacturer’s protocol. Single-stranded cDNA was synthesized with the Transcriptor First Strand cDNA Synthesis Kit. Real-time quantitative PCR (RT-PCR) was conducted to determine the expression of PPARα, cholesterol 7 alpha-hydroxylase A1 (CYP7A1), 3-hydroxy-3-methylglutaryl-coenzyme A reductase (HMGCR), adenosine monophosphate activated protein kinase (AMPK), ACC in the liver, carnitine palmitoyl transferase 1A (CPT1A), and ATP-binding cassette G5 (ABCG5), fatty acid translocase CD36 (CD36), Niemann-Pick C1-like 1 (NPC1L1), fatty acid transport protein 4 (FATP4), and microsomal triacylglycerol transfer protein (MTP) in the small intestine mRNA. Quantitative PCR primers were designed using Primer Premier 6.0 and Beacon Designer 7.8 and synthesized by Shenggong Bioengineering Co., Ltd. (Shanghai, China). RT-PCR was performed with PowerUp™ SYBRTM Green Master Mix (Applied Biosystems, Foster City, CA, USA) using CFX384 Real-Time Fluorescence Quantitative PCR System (Bio-Rad, Hercules, CA, USA). The primer sequences are shown in Table 1. The reaction conditions were as follows: 95°C for 1 min and 40 cycles of 95°C for 15 s and 63°C for 25 s to collect fluorescence. The expression of genes was normalized in reference to the housekeeping gene GAPDH, and the relative gene expression in the six groups was statistically analyzed using the 2^–ΔΔCt^ method.

**TABLE 1:** Real-time PCR primers in rat liver and small intestine.

Gene	GenBank accession	Reverse transcription primer sequences (5′–3′)	Size (bp)
Rat GAPDH	NM_017008.4	GAAGGTCGGTGTGAACGGATTTG	127
		CATGTAGACCATGTAGTTGAGGTCA	
Rat PPARα	NM_013196.2	GGAGGCAGAGGTCCGATT	131
		TCAGCAAGGTAACCTGGTCATTCAA	
Rat CPT1A	NM_031559.2	GCACATTAGACCGTGAGGAACT	138
		CCTTGATATGTTGGATGGTGTCTGT	
Rat ACC	NM_022193.1	GAGGTTGGCTATCCAGTGATGA	102
		CTGTCTGAAGAGGTTAGGGAAGT	
Rat CYP7A1	NM_012942.2	CAAGACGCACCTCGCTATTCTCT	113
		CTTCAGAGGCTGCTTTCATTGCT	
Rat HMGCR	NM_013134.2	CCTGCGTGTCCCTGGTCCTA	125
		CCTTTGGGTTACTGGGTTTGGT	
Rat AMPK	XM_008763901.1	GATTTGCCCAGTTACCTCTTTCC	156
		CACTGCGAGCTGGTCTTGA	
Rat CD36	AF072411.1	CGGTTGGAGACCTACTCATTGA	147
		CCACTTCCTCTGGGTTTTGC	
Rat FATP4	NM_001100706.1	CCTCTACCACTCAGCAGGAAA	156
		CGGCAAAGCTCACCAATGTAC	
Rat ABCG5	NM_053754.2	CTTCTGTGCCAAATAACCCAATG	135
		GGATGACAAGAGTCGGGATGAA	
Rat NPC1L1	NM_001002025.1	GCTGCTGTTTCTGACCCTGTTT	141
		CCCACTTCAAGGTATCGGTTCAG	
Rat MTP	NM_001033694.1	GTTCTCCCAGTACCCGTTCTTGGT	100
		CCTCCCTGTGGATAGCCTTTCAT	

### Western blot

Radio immunoprecipitation assay lysis. buffer including protease inhibitor cocktail (Thermo Fisher, Waltham, MA, USA) was used for the extraction of the total proteins of liver and small intestine, and the protein concentration was determined using BCA quantitative kit (Beyotime, Shanghai, China). The total extracted proteins were separated by SDS-PAGE for 2 h and then transferred to a PVDF membrane for 2 h. The membrane was incubated with T-TBS (containing 5% BSA) for 1 h and subsequently with the following primary antibodies: rabbit anti-CYP7A1 (1:1,000, Biorbyt orb539102, Cambridge, UK), anti-PPARα (1:1,000, Abcam ab126285, Cambridge, UK), anti-ABCG5 (1:1,000, Proteintech 27722-1-AP, Chicago, IL, USA), anti-NPC1L1 (1:500, Thermo Fisher PA5-72938, Waltham, MA, USA), anti-CPT1(1:500, Abcam ab128568, Cambridge, UK), and anti-GAPDH (1:10,000, Abcam ab181602, Cambridge, UK) as internal control at 4°C overnight. After being washed with T-TBS, the membranes were incubated with HRP-conjugated goat anti-mouse IgG secondary antibody (1:5,000, Thermo Pierce, Waltham, MA, USA) and goat anti-rabbit IgG secondary antibody (1:5,000, Thermo Pierce, Waltham, MA, USA) at room temperature for 1 h. Protein expression was visualized on X-ray films using SuperSignal^®^ West Dura Extended Duration Substrate (Thermo Pierce, Waltham, MA, USA). Image J 1.8.0 was used to analyze the optical density values of bands, and each test was repeated three times.

### Isolation and characterization of serum extracellular vehicles

The cell fragments in the serum were removed by centrifugation at 10,000 rpm for 30 min, and the supernatant was transferred to Beckman L-100XP Ultracentrifuge (Brea, CA, USA) at 100,000 × g for 75 min and then discarded. The precipitate was washed and resuspended with PBS for another centrifugation at 100,000 × g for 75 min (24). After centrifugation, the supernatant was discarded, and the precipitate was resuspended with 200 μl of PBS to obtain serum extracellular vehicles (EVs). Quantification was performed using BCA kits (Thermo Fisher Co., Ltd., Waltham, MA, USA). In characterizing EVs, three or more proteins must be reported in at least a semi-quantitative manner, single vesicles must be examined, and the size distribution of EVs must be measured (25). EVs were characterized by TEM HT7700, and the average EV particle size was analyzed by Flow NanoAnalyzer N30E (Xiamen Fuliu Biological Technology Co., Ltd., Xiamen, China). EV-labeled proteins tetraspanins (CD9 and CD63) and endosome or membrane-binding proteins (TSG101) were quantitatively detected by Western blot.

### QRT-PCR assay for miRNAs in serum extracellular vehicles

For the selection of miRNAs closely related to the mRNAs of lipid metabolism, the species was first selected as rats on miRDB.^1^ Several mRNAs related to lipid metabolism were then imported. One example is CYP7A1, that is, all miRNAs related to CYP7A1 gene were analyzed. For reliable results, all the miRNAs related to CYP7A1 gene obtained from the last website were inputted in another web site TargetScanMouse.^2^ Hundreds of related genes were obtained for each miRNA. CYP7A1 was searched in this list. If the results overlap, then this miRNA will be selected as an alternative. Finally, miR-21-5p, miR-30b-5p, miR-33-5p, miR-27a-3p, and miR-126a-5p were identified as possibly interacting with target genes PPARα, MTP, CYP7A1, ABCG5, and HMGCR, respectively (14).

First, serum EV miRNA was extracted as follows. EVs were added with 300 μl of binding buffer, shaken evenly, and centrifuged at 12,000 *g* for 10 min. The supernatant was transferred to a spin cartridge, and the precipitate was retained and added with anhydrous ethanol to obtain a final ethanol concentration of 70%. The mixture was transferred to the second spin cartridge and centrifuged at 12,000 *g* for 1 min. The waste was discarded, and the sample was centrifuged again at 12,000 *g* for another 1 min after 500 μl of wash buffer was added to the spin cartridge. The waste liquid was discarded, and the above steps were repeated. Idling centrifugation (12,000 g, 5 min) was performed to dry the adsorption column, and centrifugation was conducted at 12,000 *g* for 2 min. Finally, the spin cartridge was added with 50 μl of RNase-free ddH_2_O and then stored at –80°C after being placed at room temperature for 2 min (26). qRT-PCR was performed in line with the above steps. The reverse-transcription primer sequences are shown in Table 2, and the qRT-PCR primers are displayed in Table 3.

**TABLE 2:** Reverse transcription primer sequences of miRNAs.

Gene	GenBank accession	Reverse transcription primer sequences (5′–3′)
rno-mir-27a-3p	MIMAT0000799	GTCGTATCCAGTGCAGGGTCCGAGGTATTCGCACTGGATACGACGCGGAA
rno-mir-33-5p	MIMAT0000812	GTCGTATCCAGTGCAGGGTCCGAGGTATTCGCACTGGATACGACTGCAAT
rno-mir-126a-5p	MIMAT0000831	GTCGTATCCAGTGCAGGGTCCGAGGTATTCGCACTGGATACGACCGCGTA
rno-miR21-5p	MIMAT0000790	GTCGTATCCAGTGCAGGGTCCGAGGTATTCGCACTGGATACGACTCAACA
rno-miR30b-5p	MIMAT0000806	GTCGTATCCAGTGCAGGGTCCGAGGTATTCGCACTGGATACGACAGCTGA

**TABLE 3:** Real-time PCR primers of miRNAs.

Gene	Forward primer and universal primer (5′–3′)
rno-mir-33-5p-F	CGCGGTGCATTGTAGTTGC
rno-mir-27a-3p-F	GCGCGTTCACAGTGGCTAAG
rno-mir-126a-5p-F	GCGCGCATTATTACTTTTGGTACG
rno-miR21-5p-F	GCGCGTAGCTTATCAGACTGA
rno-miR30b-5p-F	GCGCGTGTAAACATCCTACAC
Universal reverse primer (micro-R)	AGTGCAGGGTCCGAGGTATT

### Double luciferase assay

293T cells in logarithmic growth phase were seeded in 24-well plates and transfected with Lipofectamine™ 3000 Transfection Reagent at approximately 60% confluence. Before transfection, the medium was replaced with a fresh one. MiRNA-30b-5p mimics and miRNA-126a-5p mimics were prepared at 20 pmol/well, and recombinant plasmid pmirGLO-MTP-WT or pmirGLO-MTP-MUT and pmirGLO-HMGCR-WT or pmirGLO-HMGCR-MUT were added at 500 ng/well. The 24-well plates with fresh medium were placed in an incubator at 37°C with 5% CO_2_ for 8 h. After 48 h, each well was washed with PBS twice and added with 250 μl of 1 × PLB lysate before the cells were lysed at room temperature. Approximately 100 μl of LAR II was absorbed in a black 96-well plate, followed by the addition of 20 μl of lysis solution. After the reading of mixture was checked, Stop & Glo substrate (100 μl) was added within 10 s and the new reading was recorded again.

### Gut microbiota composition analysis

Microbial DNA was extracted from rat cecal content samples with the Fast DNA SPIN kit (MPBIO, CA, USA) in accordance with the instructions. The V4–V5 region of the bacteria 16S ribosomal RNA gene was amplified by PCR under specific conditions (95°C for 2 min, followed by 25 cycles at 95°C for 30 s, 55°C for 30 s, and 72°C for 30 s and a final extension at 72°C for 5 min) and using primers 515 F (5′-barcode-GTGCCAGCMGCCGCGG-3′) and 907 R (5′-CCGTCAATTCMTTTRAGTTT-3′); the barcode of which has an eight-base sequence unique to each sample. Amplicons were extracted from 2% agarose gels, purified with the AxyPrep DNA Gel Extraction Kit (Axygen Biosciences, Union City, CA, USA) and quantified using QuantiFluor™-ST (Promega, Madison, WI, USA). The Purified PCR products were quantified by Qubit^®^3.0 (Life Invitrogen). The pooled DNA product was used to construct Illumina Pair-End library following Illumina’s genomic DNA library preparation steps. The amplicon library was then paired-end sequenced (2 × 250) on an Illumina Novaseq platform (Mingke Biotechnology Co., Ltd., Hangzhou, China). The image data files obtained by high-throughput sequencing were converted into sequencing reads, which were then stored in FASTQ files including their sequence information and sequencing quality information. For reliable results in subsequent analysis, the raw data were first filtered through Trimmomatic v0.33, CutAdapt 1.9.1 and custom Perl scripts were then used to identify and remove primer sequences, and high-quality reads without primer sequences were finally generated. USEARCH (version 10^3^) was employed to assign sequences with ≥ 97% similarity to the same OTUs. Tukey’s method and R software were used for the comparison of alpha diversity index between samples (*P* = 0.05). Beta diversity analysis and mapping were performed on QIIME (27) to reveal differences and similarities among the samples as measured by principal coordinate analysis (PCoA) with R software. LEfSe method was applied to compare the differential abundances of bacteria among groups at family and genus levels (28). Only those taxa with a log LDA score > 4 were considered.

### Statistical analysis

Data were shown as mean ± standard deviation (SD). Statistical differences were analyzed by one-way ANOVA with Tukey’s multiple comparison test using SPSS 18.0 (*P < 0.05* indicated significant differences).

## Results

### Characterization of NFEG-microgel

**FIGURE 1:**
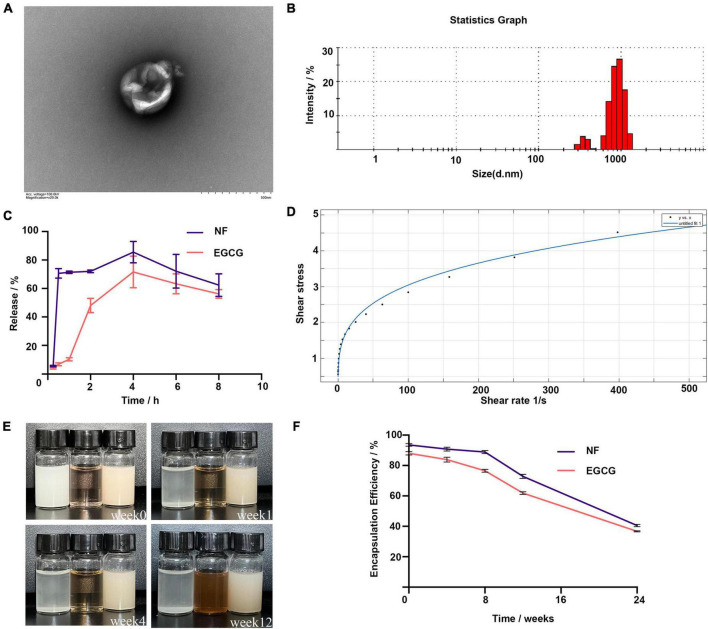
Characterization of NFEG-microgel. **(A)** NFEG-microgel observed by TEM, **(B)** average particle size of NFEG-microgel, **(C)** simulated NFEG-microgel digestion in SGF and SIF, **(D)** rheological fitting curve, **(E)** storage of NFEG-microgel at 4°C for 12 weeks (from left to right, NF solution, EGCG aqueous solution, and NFEG were shown), and **(F)** encapsulation rates during storage for 24 weeks.

The encapsulation rates of NF and EGCG by NFEG-microgel were 90 and 86%, respectively, and that of NF by liposomes was 93.5%. The average particle size, PDI, and zeta potential of NF-liposome were 104.8 nm, 0.226, and +59.8 mV, respectively, and those of NFEG-microgel were 2595 nm, 0.532, and –41.6 mV, respectively. Absolute zeta potentials greater than 30 were considered as stable. The microstructural morphologies of NFEG-microgel were observed by TEM as shown in Figure 1A. All the microgels had regular spherical or subspherical shape with neat edges. The average particle size of NFEG-microgel was 0.5∼3.0 μm (Figure 1B), which was in line with the above results. According to the rheological data, the shear viscosity of NFEG-microgel decreased with the increase in shear rate. The mathematical power-law function model was fitted to the obtained data using the cf tool in MATLAB R2020a to determine the consistency index *k* and fluidity index *n*. The fitting coefficient R^2^ was 0.99, indicating that NFEG-microgel conforms to the power-law fluid, that is, it is a non-Newtonian fluid. The *k* value was 0.899, and the *n* value was 0.265. If *n* < 1 in power-law fluid, then NFEG-microgel is a fake plastic fluid (Figure 1D).

#### Release

The NF in NFEG-microgel had a good release ability under SGF and SIF conditions with release rates of 70.6% at 0.5 h in SGF and 85.5% at 4 h in SIF. A high release rate was maintained up to 8 h. However, the release characteristics of EGCG in NFEG-microgel differed from those of NF. EGCG was released slowly in SGF and reached about 48% release rate at 2 h. Meanwhile, NFEG-microgel entered the digestive SIF, promoting the sustained release of EGCG in the microgel. The release rate reached 71.5% at 4 h and remained high up to 8 h (Figure 1C). The microgels protected EGCG and ensure its slow and sustained release because chitosan coating can increase the adhesion of mucosa.

#### Storage stability

The storage stability of NFEG-microgel was evaluated by its apparent appearance upon storage at 4°C for 12 weeks. Figures 1E,F show that even after being stored for 12 weeks, the freshly prepared NFEG-microgel still showed good stability in terms of color, uniformity, and encapsulation rate and maintained 76 and 68% encapsulation rates for NF and EGCG, respectively. Meanwhile, NF solution of the same concentration dissolved in 0.5% sodium carboxymethyl cellulose exhibited precipitation at the 1st week, indicating the conventional suspension dosage form of NF had disadvantages such as uneven dispersion and instability. The same concentration of EGCG solution exhibited discoloration at the 4th week, indicating that the stability of conventional solution was poor (Figure 1E). In conclusion, NFEG-microgel increased the water solubility, dispersion, and stability of NF and enabled its sustained and stable release. Moreover, NFEG-microgel protected the storage stability of EGCG, which is easily oxidized and discolored when placed in a complex external environment. NFEG-microgel also inhibited the direct digestion and destruction of EGCG in the intestine due to the adhesion of chitosan to intestinal mucosa.

### NFEG-microgel significantly reduced the body weight and ameliorated lipid metabolism disorder in high-fat diet rats

**FIGURE 2:**
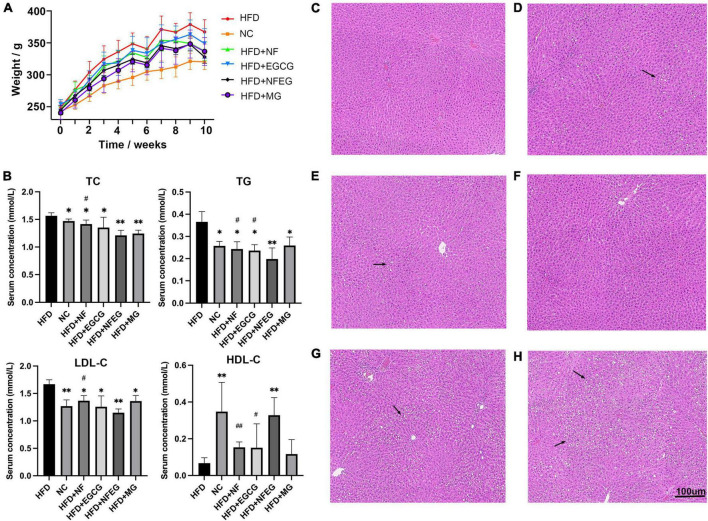
Body weight, serum biochemical indexes and H&E staining in different treatment groups: **(A)** body weight of rats, **(B)** serum TC, TG, LDL-C, and HDL-C concentrations, liver H&E staining **(C)** NC group, **(D)** HFD + NF group, **(E)** HFD + EGCG group, **(F)** HFD + NFEG group, **(G)** HFD + MG group, and **(H)** HFD group. (*P < 0.05) and (**P < 0.01) vs. HFD group; (^#^*P* < 0.05) and (^##^*P* < 0.01) vs. HFD + NFEG group.

The liver of the rat in each group was photographed (Supplementary Figure 1). In terms of appearance, the liver of rat in the HFD + NF, HFD + EGCG, HFD + NFEG, HFD + MG, and HFD groups was larger and showed different degrees of yellowing compared with that in the normal group. The liver color of the HFD + NFEG group was the closest to that in the normal group. The body weights of different groups showed an increasing trend. At the end of HFD feeding for 8 weeks, the body weight of rats was significantly higher in the HFD group than that in the NC group but lower than that in the HFD + NF, HFD + EGCG, HFD + NFEG, and HFD + MG groups. The HFD + NFEG group showed the most remarkable reduction in body weight (Figure 2A). The total 8-week feed consumption in kilograms (kg) for the NC, HFD + NF, HFD + EGCG, HFD + NFEG, HFD + MG, and HFD groups was 5.67, 5.31, 5.04, 5.61, 5.51, and 5.69 kg, respectively. According to these data, the normal diet consumption per day was consistent with the rising trend of body weight gain in the NC group. A slight decrease in HFD consumption per day was observed in the other groups from the 5th week to the last week. The trends of water intake per day were in accordance with the diet consumption, and no difference in weekly water consumption was found among the groups.

As shown in Figure 2B, the NC, HFD + NF, HFD + EGCG, HFD + NFEG, HFD + MG groups had significantly lower serum TC, TG, and LDL-C concentrations (*P <* 0.05) and significantly higher HDL-C content (*P <* 0.05) than the HFD group, except for the HFD + MG group whose HDL-C content showed no significant difference from that in the HFD group. The difference between HFD + NFEG and HFD groups was the most significant (*P <* 0.01). In particular, the HFD + NFEG group had significantly lower TC and LDL-C contents than the HFD + NF group and significantly different TG and HDL-C contents compared with those in the HFD + NF and HFD + EGCG groups (*P <* 0.05).

Rat liver H&E staining results showed that the hepatocytes filled with large lipid composition had balloon-like changes, and the hepatocyte nuclei were constricted and deformed in the HFD group. Meanwhile, the amounts of lipid droplets and the degree of liver fat cavitation in the HFD + NF, HFD + EGCG, and HFD + NFEG groups were reduced compared with those in the HFD group, indicating that HFD-induced hepatic fat accumulation could be reduced by NF and EGCG (Figures 2C–H). These results demonstrated that NFEG-microgel has a better effect on improving the lipid profile, hepatic steatosis, and liver injury than HFD or feeding with single package NF or EGCG-microgel.

### NFEG-microgel significantly regulated the mRNAs in liver and small intestine

**FIGURE 3:**
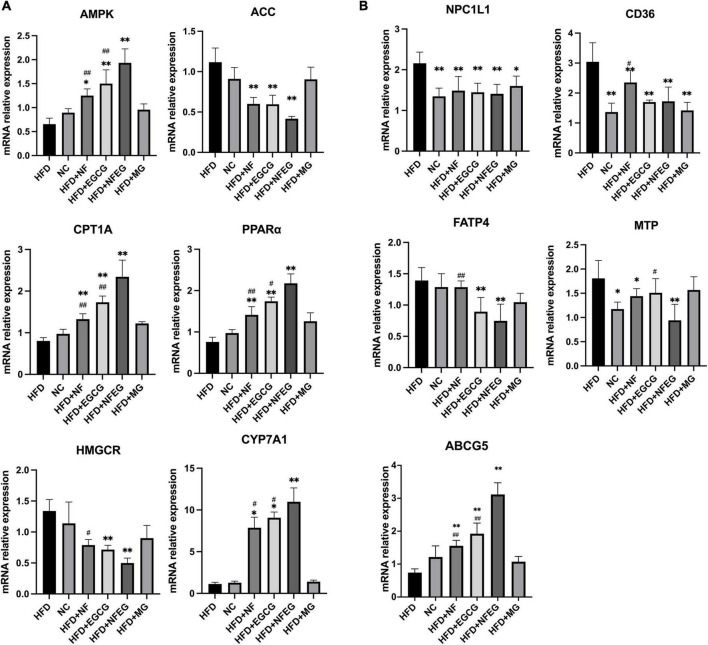
mRNA levels in rat liver and small intestine: **(A)** expression of AMPK, ACC, CPT1A, PPARα, HMGCR, and CYP7A1 in rat liver; and **(B)** expression of NPC1L1, CD36, FATP4, MTP, and ABCG5 in rat small intestine. (*P < 0.05) and (**P < 0.01) vs. HFD group; (^#^*P* < 0.05) and (^##^*P* < 0.01) vs. HFD + NFEG group.

Key genes associated with lipid metabolism in the liver and small intestine were detected, and differences were observed among the groups. HFD + NFEG had significant effects on liver PPARα, AMPK, ACC, CYP7A1, CPT1A, and HMGCR (*P <* 0.01) compared with HFD. The expression of AMPK, CPT1A (*P <* 0.01), and CYP7A1 (*P <* 0.05) genes in the HFD + NFEG group were significantly higher than those in the HFD + NF and HFD + EGCG groups (Figure 3A). HFD + NFEG downregulated the expression of NPC1L1, MTP, CD36, and FATP4 and significantly repressed the downregulation of ABCG5 in the small intestine of HFD rats (*P <* 0.01). The HFD + NFEG group had significantly higher expression of ABCG5 gene (*P <* 0.01) and significantly lower expression of MTP genes (*P <* 0.05) compared with the HFD + NF and HFD + EGCG groups. FATP4 expression in the HFD + NFEG group was significantly decreased compared with that in the HFD + NF group (*P <* 0.01). CD36 and FATP4 expression showed no significant differences between the HFD + EGCG and HFD + NFEG groups, indicating that EGCG mainly inhibited lipid absorption after its release in the intestine. In addition, the expression of NPC1L1 and CD36 in the HFD + MG group was as low as that in the HFD + NFEG group (Figure 3B), indicating that the blank microgel had a similar effect on lipid absorption.

### NFEG-microgel significantly regulated the protein expression of genes in liver and small intestine

**FIGURE 4:**
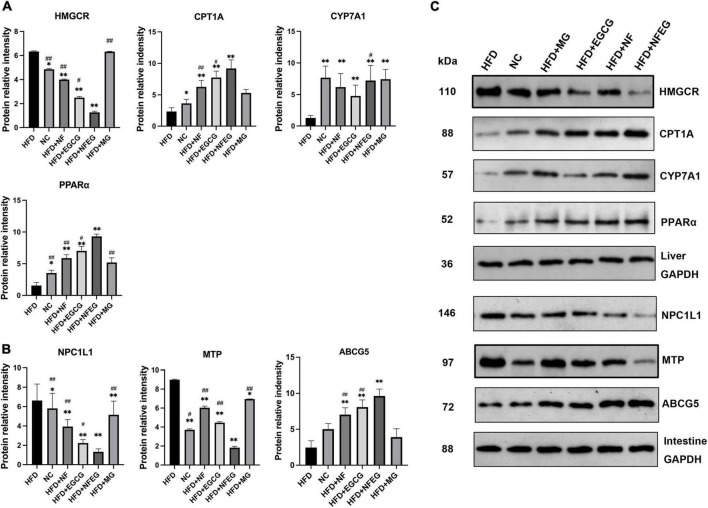
Protein expression of genes in the liver and small intestine; the results of Western blot were almost consistent with those of qRT-PCR. **(A)** Protein relative intensity expression of HMGCR, CYP7A1, CPT1A, and PPARα in rat liver. **(B)** Protein relative intensity expression of NPC1L1, MTP, and ABCG5 in rat small intestine. **(C)** WB bands. (*P < 0.05) and (**P < 0.01) vs. HFD group; (^#^*P* < 0.05) and (^##^*P* < 0.01) vs. HFD + NFEG group.

Western blot was used to detect liver PPARα, CYP7A1, HMGCR, and CPT1A and small intestine NPC1L1, MTP, and ABCG5. As shown in Figure 4, differences in the expression of these proteins were observed among the groups. The protein expression levels of PPARα, CYP7A1, and CPT1A were significantly higher in the HFD + NF, HFD + EGCG, and HFD + NFEG groups than in the HFD group (*P <* 0.01) and were higher in the HFD + NFEG group than in the HFD + NF and HFD + EGCG groups (*P <* 0.05). The protein expression level of HMGCR was significantly higher in the HFD and HFD + MG groups than other groups (*P <* 0.05) where that in the HFD + NFEG group was the lower than others (*P <* 0.05). The protein expression levels of NPC1L1 and MTP significantly decreased in the HFD + NF, HFD + EGCG, and HFD + NFEG groups compared with those in the HFD group (*P <* 0.01) and were lower in the HFD + NFEG group than in the NC, HFD + NF and HFD + EGCG groups (*P <* 0.05). The protein expression levels of ABCG5 were significantly higher in the HFD + NF, HFD + EGCG, and HFD + NFEG groups than in the HFD group (*P <* 0.01) and were higher in the HFD + NFEG group than in the HFD + NF and HFD + EGCG groups (*P <* 0.01). Meanwhile, the protein expression levels of PPARα, CPT1A, HMGCR, NPC1L1, and ABCG5 in the HFD + MG group showed no significant differences from those in the HFD group (*P >* 0.05), except for NPC1L1 and MTP (*P <* 0.05).

### Identification of extracellular vehicles in the serum

**FIGURE 5:**
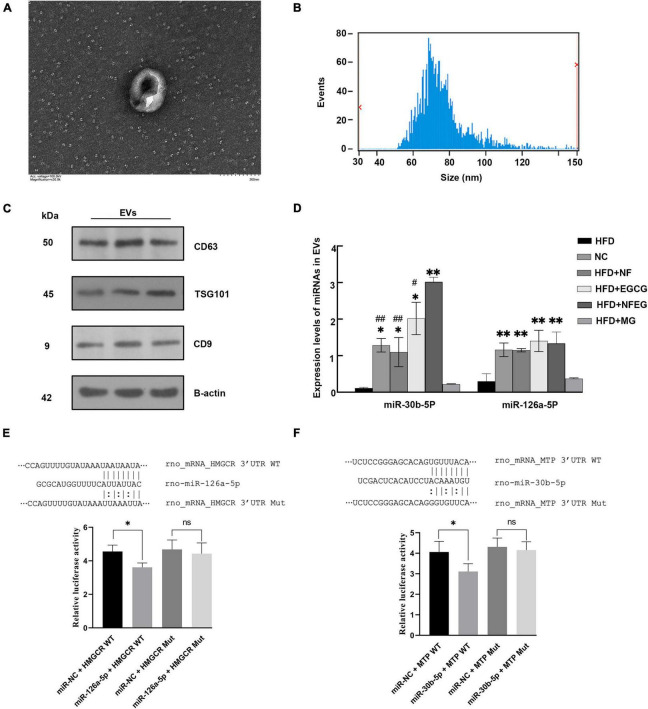
Identification and miRNA expression level of EVs and dual-luciferase reporter assay analysis. **(A)** Serum EVs of different groups observed by transmission electron microscopy (TEM) at the scale of 200 nm. **(B)** Particle size analysis showing that the particle size of each exosome ranged from 50 to 140 nm. **(C)** Expression levels of CD9, CD63, and TSG101 determined by Western blot. **(D)** miRNAs were extracted from rat serum EVs, and their expression levels were detected by qRT-PCR. Significant difference was indicated with the sign (*P < 0.05) and (**P < 0.01) vs. HFD group; (^#^*P* < 0.05) and (^##^*P* < 0.01) vs. HFD + NFEG group. Dual-luciferase reporter assay analysis: **(E)** the top portion of panel **(E)** shows the binding site of miR-126a-5p mimics and corresponding target genes HMGCR, and the bottom portion of panel **(E)** shows the relative fluorescence values after transfection and mutation, which suggested that significant interaction existed between miR-126a-5p and target gene HMGCR. Panel **(F)** shows similar results on the interaction of miR-30b-5p with MTP. Significant difference was indicated with the sign *P < 0.05.

As shown in the TEM observation in Figure 5A, the serum EVs had spherical structures and saucer shape. Their particle size range within 50–140 nm (Figure 5B), and the average particle sizes of the NC, HFD + NF, HFD + EGCG, HFD + NFEG, HFD + MG, and HFD groups were 86.92, 83.71, 88.08, 96.01, 92.45, and 84.69 nm, respectively. For EV characterization, three marker proteins were reported in at least a semi-quantitative manner. Western blot analysis showed that the expression levels of the surface proteins of EVs, CD9, CD63, and TSG101 were high in the EVs (Figure 5C).

### NFEG-microgel significantly regulated the level of miRNA in serum extracellular vehicles

Five miRNAs closely associated with lipid metabolism were detected. Compared with those in the HFD and HFD + MG groups, the expression levels of miR-30b-5p and miR-126a-5p in the NC, HFD + NF, HFD + EGCG and HFD + NFEG groups were significantly increased (*P < 0.05*). No significant difference was observed in the expression level of miR-21-5p, miR-33-5p, and miR-27a-3p among the groups. In addition, the expression level of miR-30b-5p in the HFD + NFEG group was higher than that in the NC, HFD + NF, and HFD + EGCG groups (*P < 0.05*), and the expression level of miR-126a-5p showed no significant differences among the NC, HFD + NF, HFD + EGCG, and HFD + NFEG groups (Figure 5D). In accordance with the qRT-PCR results of lipid metabolism genes in the liver and small intestine, miR-126a-5p and miR-30b-5p may interact with their target genes HMGCR and MTP, respectively.

### Effects of NFEG-microgel on dual-luciferase assay

qRT-PCR results elucidated that the expression levels of miR-126a-5p and miR-30b-5p in the serum EVs significantly differed among the six treatment groups and showed negative correlation with the expression of HMGCR and MTP. TargetScan and miRDB databases predicted possible interaction sites between miR-126a-5p and miR-30b-5p and their target genes HMGCR and MTP mRNA, respectively. The interactions of miR-30b-5p-MTP and miR-126a-5p-HMGCR were further tested by constructing the reporter plasmids of the potential action points of HMGCR and MTP and conducting dual-luciferase reporter assay.

As shown in Figures 5E,F, the relative fluorescence value of rno-miR-126a-5p + HMGCR-WT co-transfection group were lower (*P < 0.05*) than that of miR-NC + HMGCR-WT co-transfection group. After HMGCR mRNA mutation, no difference in relative fluorescence value was observed between the co-transfections of miR-NC+HMGCR and rno-miR-126a-5p + HMGCR groups (*P > 0.05*; Figure 5E). Dual-luciferase reporter assay results of miR-30b-5p and MTP showed similar results (Figure 5F). The above results implied the existence of interaction sites for miR-126a-5p and miR-30b-5p and their respective target mRNAs, HMGCR and MTP. These sites may be the key to relieve NAFLD by post-transcription mechanism between miRNAs and mRNAs.

### NFEG-microgel altered the composition and diversity of gut microbiota in high-fat diet rats

**FIGURE 6:**
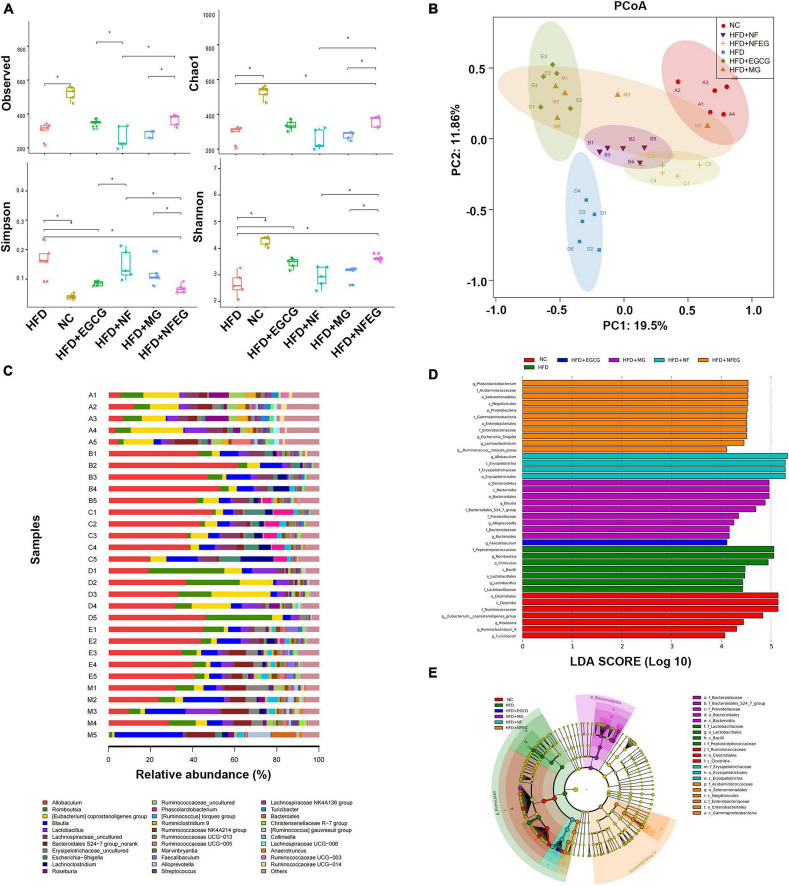
NFEG-microgel changed the diversity and composition of gut microbiota in HFD-fed rats. **(A)** α-diversity index (observed, Chao1, Shannon, and Simpson index). **(B)** Bray–Curtis PCoA plot based on the OTU abundance of each rat. **(C)** Heat map of bacterial taxonomic profiling at the genus level of intestinal bacteria based on each rat from different groups: NC group (A1–A5), HFD + NF group (B1–B5), HFD + NFEG group (C1–C5), HFD group (D1–D5), HFD + EGCG group (E1–E5), and HFD + MG group (M1–M5). Linear discriminant analysis (LDA) scores **(D)** and cladogram **(E)** generated from linear discriminate analysis effect size (LEfSe) analysis, showing the biomarker taxa (LDA score of > 4 and a significance of *P* < 0.05 determined by the Wilcoxon signed-rank test). Significant difference was indicated with the sign *P < 0.05 and ^**^P < 0.01.

**FIGURE 7:**
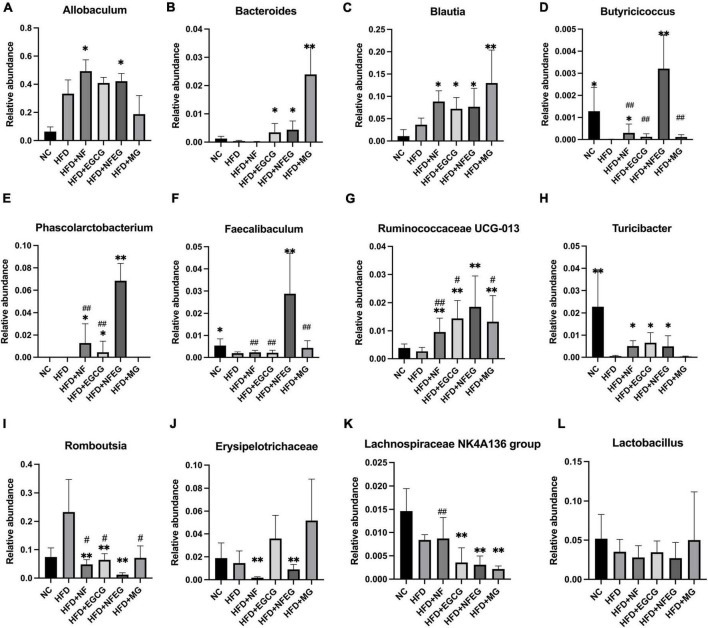
NFEG-microgel changed the relative abundance of gut microbiota in HFD-fed rats. Relative abundance of *Allobaculum* **(A)**, *Blautia* **(B)**, *Bacteroides* **(C)**, *Butyricicoccus* **(D)**, *Phascolarctobacterium* **(E)**, *Faecalibaculum* **(F)**, *Ruminococcaceae UCG-013* **(G)**, *Turicibacter* **(H)**, *Romboutsia* **(I)**, *Erysipelotrichaceae* **(J)**, *Lachnospiraceae_NK4A136_group* **(K)**, and *Lactobacillus* **(L)**. (*P < 0.05) and (**P < 0.01) vs. HFD group; (^#^*P* < 0.05) and (^##^*P* < 0.01) vs. HFD + NFEG group.

The gut microbiota may be an underlying target to treat obesity and related metabolic diseases. Bacterial 16S rRNA in stool was conducted to examine whether NFEG-microgel can alter the composition of the gut microbiota. Analysis of α-diversity index (Chao1 index, Shannon index, and Simpson index) reflecting gut microbiota showed that α-diversity increased with the increase in Chao1 index and Shannon index and the decrease in Simpson index (Figure 6A). Significant differences were observed among the HFD + NFEG and HFD, HFD + MG, and HFD + NF groups. Bray–Curtis PCoA analysis of OTU abundance of each rat revealed that compared with that in the NC group, the intestinal microbiota in the HFD group showed significant structural changes along the first principal component (PC1) and the second principal component (PC2). Compared with the NC group, the HFD + MG and HFD + EGCG groups showed the same shift as the HFD group along PC1 whereas the HFD + NFEG group had almost the same intestinal microbiota as the NC group along both PC1 and PC2 (Figure 6B). At the genus level, high-abundance species were selected to profile the expression of each group with the heatmap in Figure 6C. From a homoplastic viewpoint, linear discriminant analysis effect size (LEfSe) method was employed to identify statistically significant biomarkers and dominant microbiota among these groups (Figures 6D,E). Unexpectedly, Firmicutes was found to be the primary phylum of the gut microbiota in the HFD group, and this finding agreed with the study of Wang et al. (5). Meanwhile, Bacterioidetes was confirmed to be the dominating phylum of the gut microbiota in the HFD + MG group. The relative abundance of *Allobaculum, Blautia, Bacteroides, Butyricicoccus, Phascolarctobacterium, Faecalibaculum, Ruminococcaceae UCG-013*, and *Turicibacter* significantly increased (*p* < 0.05), and that of *Romboutsia, Erysipelotrichaceae*, and *Lachnospiraceae_NK4A136_group* significantly decreased (*p* < 0.01). No regulatory effect on *Lactobacillus* was found (Figures 7A–L). Experimental results showed that NF had a significant effect on *Allobaculum* (Figure 7A) and *Erysipelotrichaceae* (Figure 7J) because it only induced significant changes in the HFD + NFEG and HFD + NF groups. NFEG-microgel significantly enriched *Bacteroides, Butyricicoccus, Phascolarctobacterium, Faecalibaculum*, and *Ruminococcaceae UCG-013* and significantly reduced *Romboutsia* (*P* < 0.01) as which shown in Figures 7B,D–G,I. The relative abundances of these genera in the HFD + NFEG were significantly different from those in the HFD + NF, HFD + EGCG, HFD + MG, and HFD groups (*P* < 0.05).

## Discussion

Lipid metabolism involves the regulation cycle of multi-tissues, multi-genes, and multi-molecules. Therefore, microgels must contain multiple active components to improve the synergistic lipid-lowering effect. Elucidate the possible mechanism of lipid metabolism regulation from multiple ways is necessary. Chitosan is the only positively charged edible food fiber found in nature, and MLPE are negatively charged. Thus, these two can self-assemble and combine into stable microgels through electrostatic interaction. Microgel is mainly used to coat hydrophilic compounds. In our study, we first loaded NF into liposomes with hydrophilic surface and then incorporated the liposomes into the microgels, thus allowing the microgels to encapsule hydrophobic compounds. The HFD + NFEG group had significantly lower body weight, serum TC, TG, and LDL-C and significantly higher HDL-C compared with the HFD + NF and HFD + EGCG groups, proving that NF and EGCG were jointly involved in lipid regulation in the HFD rats. Most of the current research focuses on a single material carrier. The microgel prepared by using two natural materials in this study is safe, non-toxic, and has a certain lipid-lowering effect. Its preparation process is simple, and it has the advantages of embedding a variety of nutrients, which has potential application prospects in the field of food or nutrition.

Lipid accumulation *in vivo* is mainly related to the expression of lipid synthesis genes (such as HMGCR), lipid metabolism genes (such as PPARα, AMPK, ACC, CPT1A, and CYP7A1) in the liver, and lipid import genes (such as NPC1L1 and CD36) and lipid transporter genes (such as MTP, ABCG5, and FATP4) in the small intestine. Metabolism is regulated by reducing substrate overload to minimize the intake and transfer of metabolic substrates to metabolically active tissues. AMPK plays a central role in lipid metabolism by regulating the downstream gene ACC and CPT1 pathways. ACC catalyzes the production of malonyl-CoA, which is a major component of de novo adipogenesis and an allosteric inhibitor of CPT1 (key restriction enzyme in fatty acid β-oxidation and is also regulated by PPARα) (29). In our study, NFEG-microgel significantly increased AMPK level, decreased ACC level, and ultimately increased CPT1A function. The PPARα subtype performs its major functions in the liver, and the receptor is involved in all aspects of lipid metabolism including fatty acids (FA) transport, binding, absorption, synthesis, and oxidative degradation (30). HMGCR is a rate-limiting enzyme in cholesterol biosynthesis pathway, and CYP7A1 is a rate-limiting enzyme in the bile acid synthesis pathway; both play an important role in regulating the amount of cholesterol because bile acids provide an important excretory pathway for cholesterol metabolism (31). NPC1L1 mediates the intestinal uptake of dietary and biliary cholesterol. CD36 and FATP4 are major player in metabolic tissues and are among the proteins involved in FA uptake (32, 33). In this work, EGCG reversed the HFD-induced effects on intestinal substrate transporters CD36 and FATP4, and this finding was the same as that reported by Lu et al. (34). We found that NF and EGCG played a synergistic role in alleviating lipid accumulation. In particularly, NF mainly acted on lipid metabolism in the liver and regulated lipid transport and efflux in the intestine, and EGCG and blank microgel inhibited lipid absorption in the intestine.

As a series of post-transcriptional gene repressors, miRNAs are widely related to the regulation of gene expression, including almost all aspects of the system controlling metabolism. In regulating gene expression, miRNAs play an important role in communication. Yan et al. testified that the disruption of miR-126a in mice caused hepatocyte senescence, inflammation, and metabolism deficiency and revealed that the administration of miR-30b-5p antagonist attenuated liver inflammation in the injured liver. Zhang et al. (35) reported the increased expression of PPARα and decreased expression of lipid synthesis related gene SREBP-1 in miR-30b-5p overexpressed Huh-7 cells. Our detection results showed that the expression levels of miR-126a-5p and miR-30b-5p significantly differed among the six groups. Dual-luciferase reporter gene assay validated that miR-126a-5p and miR-30b-5p significantly interacted with their target genes HMGCR and MTP, respectively. The present study revealed that the miRNAs in the serum EVs target the genes associated with lipid metabolism as shown in the Graphical Abstract by Biorender.^4^ Therefore, the NAFLD relieving mechanism of NFEG-microgel might be related to the regulation of miRNA signaling.

Gut microbes are considered “new organs” and play an important role in metabolic disorders. Wang et al. (5) demonstrated that the abundance of Lachnospiraceae_NK4A136_group and Erysipelotrichaceae decreased after supplementation, and this finding is consistent with our experimental results. Multiple studies revealed that Turicibacter was negatively correlated with inflammation in obesity (36, 37). Kaakoush clarified a strong association between Erysipelotrichaceae and host lipid metabolism. Wang et al. (38) revealed that HFD can reduce the abundance of *Lactobacillus, Faecalibaculum*, and *Blautia*. SCFAs may contribute to energy expenditure and appetite regulation. In addition to their role in gut health and signaling molecules, SCFAs may also affect substrate metabolism and peripheral tissue function by entering the systemic circulation. A growing body of evidence proves the benefits of SCFAs in adipose tissues and liver substrate metabolism and function (39). Zhang et al. confirmed that berberine can significantly enrich *Blautia* and *Allobaculum*, which produce SCFAs in the intestine, and consequently increase the amount of intestinal SCFAs (40).

According to the outcomes of PCoA and stratified cluster analysis, HFD feeding profound altered the composition and diversity of the gut microbiota, and NFEG-microgel significantly improved the intestinal microbiota dysbiosis in rats. NFEG-microgel significantly enriched the bacteria that produce SCFAs, including *Allobaculum, Blautia, Bacteroides, Butyricicoccus, Phascolarctobacterium* (41), *Romboutsia*, and *Faecalibaculum* (42), suggesting that these SCFA-producing bacteria play an important role in the efficacy of NFEG-microgel.

However, we only broadly illustrated the association, but not the causality, between the improved gut microbiota and the anti-obesity effects of NFEG-microgel. Further studies with feces transplantation experiments are necessary to clarify this association. Additionally, chitosan and proanthocyanidins were used to prepare microgels, which proved to be stable and effective, but the molecular mechanism of their binding still needs to be further explored. In addition, the source and production mechanism of EVs need further study.

In conclusion, NFEG-microgel could prominently inhibit the development of NAFLD and its related metabolic alterations by suppressing body weight gain, reducing serum lipids, ameliorating hepatic injury, and regulating the genes associated with lipid metabolism and miRNA increase. Furthermore, NFEG-microgel increases the diversity of gut bacterial community and the relative contents of beneficial bacteria and decreases the abundance of pathogenic bacteria. This generalizable strategy of encapsulating multiple nutrients in porous microgels using chitosan and MLPE can be applied in other systems to achieve the synergistic effects of multiple nutrients. NFEG-microgel offers an effective and innovative treatment of dyslipidemia.

## Data availability statement

The original contributions presented in this study are publicly available. This data can be found here: https://www.ncbi.nlm.nih.gov/bioproject/PRJNA882230.

## Ethics statement

This animal study was reviewed and approved by the Laboratory Animal Ethics Committee from China Jiliang University under the approval number: 2022-005.

## Author contributions

SZ designed the experimental scheme, performed the experiment operation, analyzed the data, and finished the manuscript. WX completed parts of experiments including oral administration and took samples of the rats. JG guided and supervised the whole experimental process. JL, FG, AX, and JZ also provided some guidance and reviewed the manuscript. All authors contributed to the article and approved the submitted version.

## Footnotes

1
http://www.mirdb.org

2
https://www.targetscan.org/vert_80/

3
http://drive5.com/uparse/

4
http://www.biorender.com

## Supplementary material

Supplementary Figure 1 Morphological observation of liver.
