# Supplementary figures and images for: Preparation of microgel co-loaded with nuciferine and epigallocatechin-3-gallate for the regulation of lipid metabolism
Source: Front Nutr. 2022 Dec 12;9:1069797. doi: 10.3389/fnut.2022.1069797 (PMC9790983; doi:10.3389/fnut.2022.1069797)

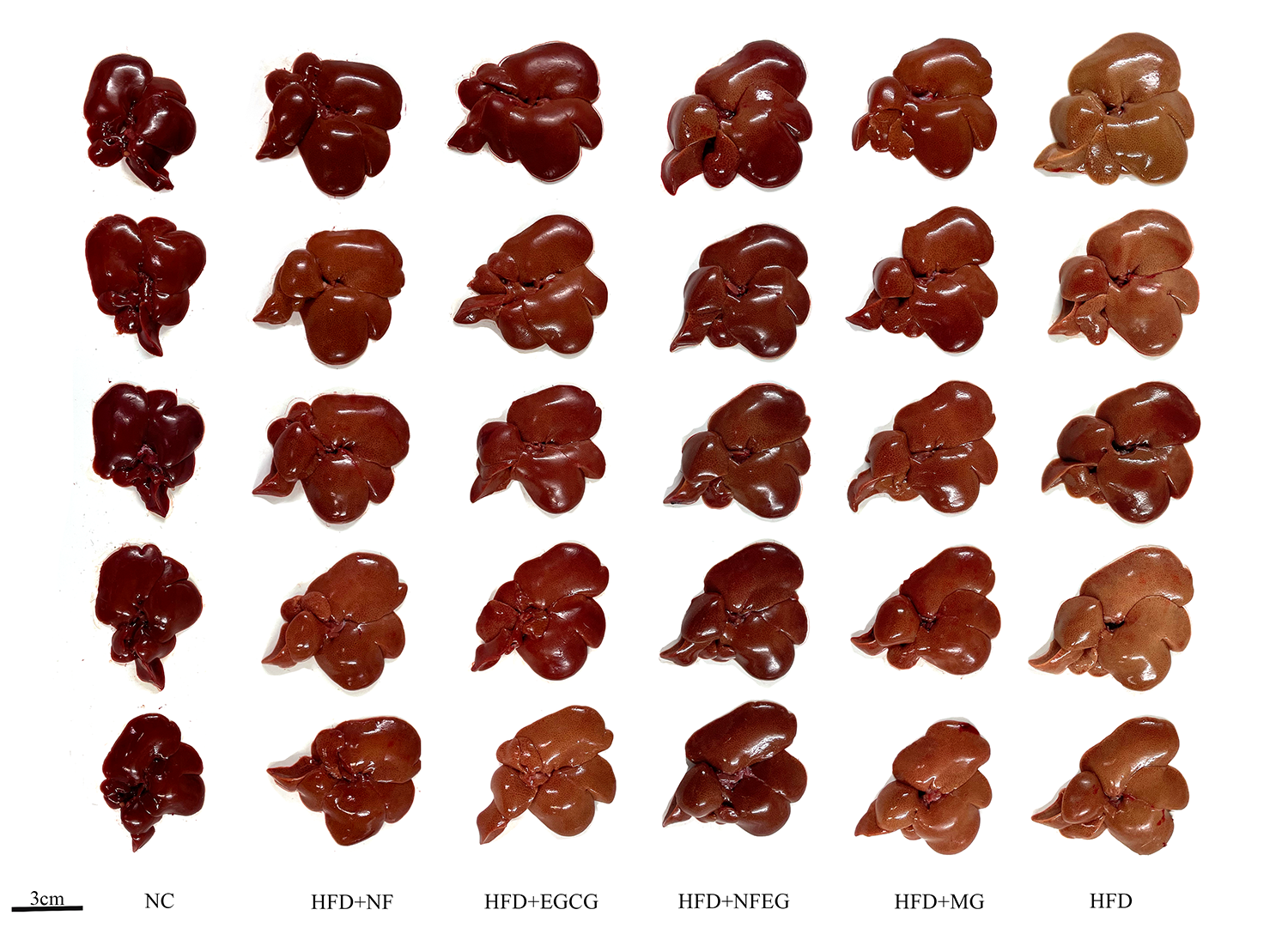

Supplement: Supplementary Figure 1 — Morphological observation of liver. [file Image_1.TIF]
